# Identifying Moderators in the Link Between Workplace Discrimination and Health/Well-Being

**DOI:** 10.3389/fpsyg.2020.00458

**Published:** 2020-03-17

**Authors:** Yue Ethel Xu, William J. Chopik

**Affiliations:** Department of Psychology, Michigan State University, East Lansing, MI, United States

**Keywords:** workplace discrimination, health and retirement study, personality, workplace support, health and well-being

## Abstract

The stress that arises from workplace discrimination can have a large impact on an employee’s work attitude, their work and life satisfaction, and oftentimes whether or not they stay in a job. Workplace discrimination can also have a considerable influence on employees’ short- and long-term health. However, less is known about the factors that might mitigate or exacerbate the effects of discrimination on health. The current study focused not only on the links between workplace discrimination and health, and but also on the effects of potential moderators of the discrimination-health link (i.e., perceived control, Big Five personality traits, optimism, and coworker/supervisor support). People with high neuroticism, high extraversion and high agreeableness were more negatively affected by workplace discrimination than those low on neuroticism, extraversion, and agreeableness. Perceived control was found to be a protective factor, such that those high in perceived control had fewer chronic illnesses in the context of high levels of workplace discrimination.

## Identifying Moderators in the Link Between Workplace Discrimination and Health/Well-Being

Employees can face workplace discrimination from a variety of factors, such as their age, gender, and race. The stress that arises from workplace discrimination can have a large impact on an employee’s work attitude, their work and life satisfaction, and oftentimes whether or not they stay in a job. Workplace discrimination can also have a considerable influence on employees’ short-term and long-term health. However, less is known about the factors that might mitigate or exacerbate the effects of discrimination on health. The current study focused not only on the links between workplace discrimination and health, and but also on the effects of potential moderators of the discrimination-health link (i.e., perceived control, Big Five personality traits, optimism, and coworker/supervisor support).

### Workplace Discrimination and Health and Well-Being

Although overt, explicit display of discrimination in today’s society are rare, there are still a lot of subtle ways in which vulnerable groups are discriminated against (Carter and Murphy, 2015). Perceived discrimination is often defined as an individual’s perception of receiving (negative) differential treatment based on some characteristics (e.g., age, gender, race/ethnicity). These perceptions can have large implications for individual’s lives. Perceived discrimination has been linked to numerous physical health problems (e.g., hypertension; Pascoe and Smart Richman, 2009), unhealthy behaviors (e.g., alcohol and substance abuse; Yen et al., 1999; Martin et al., 2003; Bennett et al., 2005), and poorer health management (Yoshikawa et al., 2004). Discriminatory experiences affect health primarily through the activation of physiological stress systems and can predicts higher systolic blood pressure over the day (Pascoe and Smart Richman, 2009).

According to social identity theory, people affiliate with others who are like them because they share an emotional involvement in common identities (Tajfel et al., 1979). Because gender and race are social identities, individuals are most likely to identify and favor same-gender or same-race others. When individuals perceive that they are being treated unfairly or in a hostile way, they often attribute this poor treatment to these social identity categories. Perceived gender and race discrimination thus continue to be an issue in the workplace (e.g., personnel selection; Graves and Powell, 2008). Though pervasive and blatant prejudice decreases as social norms and laws change, the prevalence of subtle bias has *increased* according to some scholars (Carter and Murphy, 2015). In the workplace, such modern forms of discrimination can be very stressful for people from vulnerable groups. Perceived racism is related to experiences of anger and also anger suppression. Its influence during the day might result in individuals remaining in a persistent state of distress (Brondolo et al., 2008). Previous research has also found high levels of race discrimination are related to higher blood pressure, supporting this argument (Steffen et al., 2003). These physiological responses that result from stressful experiences over time can increase an individual’s susceptibility to physical illness (Pascoe and Smart Richman, 2009).

#### Perceived Discrimination

Workplaces are often diverse, comprising of individuals that differ not only in social identities like race and gender but also age. Perceived discrimination, especially toward older employees in the workplace, is a common phenomenon. Older employees are often stereotyped as lacking creativity, having poor performance, being stubborn with rules and standards, and less likely to adapt to new things and environments (Marchiondo et al., 2017). These assumptions are reflected in biased assessments from their supervisors on their job performance and economic worth, which can lead to fewer job opportunities for training and promotion. Greater wear and tear arises from older adults, struggles to cope with these negative interactions (Charles, 2010). According to the strength and vulnerability integration model (SAVI; Charles, 2010), discrimination (and other stressors) overwhelms an individual’s ability to regulate stress and therefore triggers harmful physiological responses among older adults. In Marchiondo et al. (2017) empirical test of SAVI model, perceiving more discrimination over time was associated with higher levels of depression, lower levels of job satisfaction, and poorer health among older adults.

Taken together, the results of these studies and many more suggest that perceived discrimination can have negative effects on the mental and physical health of individuals (Yen et al., 1999; Martin et al., 2003; Steffen et al., 2003; Yoshikawa et al., 2004; Bennett et al., 2005; Pascoe and Smart Richman, 2009; Marchiondo et al., 2017).

### Potential Moderators of the Link Between Perceived Discrimination and Health

Although there is substantial evidence to suggest an association between perceived discrimination and worse physical and mental health, there are likely individual difference factors that affect this link. But why might we expect to see psychological and situational characteristics moderate the perceived discrimination—health link? There are a number of theoretical reasons to expect that this link would not be uniform across individuals (Triana et al., 2015). For example, a number of theories positing interactions between personality and situations to predict adjustment can provide several insights into how personality might affect responses to workplace discrimination. Specifically, trait activation theory—and its close cousins of personality-job fit theory and person-environment interactionism—posit that certain job environments and characteristics are amenable and productive for individuals with certain personality traits (Holland, 1997; Edwards et al., 1998; Tett and Guterman, 2000; Tett and Burnett, 2003; Fleeson, 2004; Tett et al., 2013). In other words, people tend to “fit” or “not fit” psychologically with their environments.

However, it may not be normative that any individual “fits” with an environment that is particularly hostile, such as those with large amounts of discrimination. Related to this, coping theory—specifically as it relates to coping with discrimination and stigma (Berjot and Gillet, 2011)—can also provide insights into how individuals fare when faced with difficult work environments (Lazarus and Folkman, 1984; Folkman et al., 1986). In their review of the applications of the transactional model of stress and coping in the context of perceived discrimination, Berjot and Gillet (2011) specifically highlight many personal characteristics that affect the primary components of the coping process—cognitive appraisals and coping. For example, having a sense of control and autonomy over one’s environment and lower levels of anxiety are particularly effective in coping with perceived discrimination (Judge et al., 2002, 2003; Berjot and Gillet, 2011). They also identified situational characteristics—including perceiving members of your immediate environment as being supportive—as additional factors affecting the link between perceived discrimination, stress, and health. In other words, when an individual perceives that they are being discriminated against, there are a number of personal characteristics that might buffer or exacerbate the negative effects of perceived discrimination. In the current study, we also examined the roles of situational factors (e.g., coworker and supervisor support) as well.

Indeed, in the many moderating factors we examined in the current study, each have been linked to personality-situation fit theories and the transactional model of stress and coping (e.g., Riolli et al., 2002; Afshar et al., 2015). Intuitively, extensions of these models flow well to suggest that a myriad of factors would moderate the effects of discrimination on health and well-being over time in the current study. For example, some individuals might be particularly vulnerable to the effects of perceived discrimination and may experience worse outcomes as a result. Such findings are also seen in diathesis-stress demonstrations of enduring vulnerabilities paired with particularly stressful environments (Ingram and Luxton, 2005). Yet others may be less affected by perceived discrimination. Part of this differential susceptibility might arise from characteristics of individuals (e.g., their personality). Part of this differential susceptibility might arise from situational characteristics (e.g., workplace support). In the current study, we focus on a select number of individual difference and situational characteristics that might serve protective functions in the link between discrimination and health.

Worth noting, few reviews exist examining moderators of this link. The few exceptions highlight that many of the efforts to examine moderators look at constructs in isolation of one another (i.e., only one at a time) and focus on constructs less theoretically linked to perceived discrimination and health (e.g., self-esteem; Fischer and Shaw, 1999; Triana et al., 2015). Other approaches take an amalgam of possible moderating factors and consider them to be a single outcome of this link instead (i.e., “psychological health”; Pascoe and Smart Richman, 2009; Triana et al., 2015). Put plainly, there has not previously been a thorough treatment of multiple moderators examined simultaneously. In the current study, we redress this gap by examining several moderating factors previously linked to job, psychological, and health outcomes simultaneously. Specifically, we focus on Big Five personality traits, optimism, perceived control, and coworker/supervisor social support. In the sections below, we focus on the evidence linking each of these factors to perceived discrimination, coping, and health.

#### Big Five Personality Traits

Personality is considered to be characteristic and dispositional patterns in thoughts, feelings, and behaviors. Personality is commonly conceptualized and measured in the context of the Big Five personality traits: extraversion (traits like *outgoing* and *lively*), agreeableness (traits like *helpful* and *sympathetic*), conscientiousness (traits like *responsible* and *hardworking*), neuroticism (traits like *sensitive* and *moody*) and openness to experience (traits like *curious* and *imaginative*) (John et al., 2008). Perceived discrimination can cause a lot of stress to an individual (Pascoe and Smart Richman, 2009). This stress may be a temporary discomfort; but overtime, it could lead to long-term problems. People have different coping styles for how they react and make choices when they are facing difficult situations. Personality traits are shown to reliably predict coping styles and stress levels (from the transactional model of stress and coping) and specific hypotheses can be derived from each personality trait (see Afshar et al., 2015). People who score high on neuroticism experience and perceive more stressful events and negative emotions. Highly neurotic people also utilize ineffective coping strategies and often engage in avoidant coping (e.g., minimizing/ignoring stress). Conversely, openness and conscientiousness are positively associated with reinterpretation, problem-focused coping, and growth. Agreeableness is positively related to social support seeking, active coping, and negatively associated with self-blame (Afshar et al., 2015).

Personality not only affects stress-coping mechanisms, but also influences the appraisal of situations in everyday life (Vollrath, 2001). Research has shown that Big Five personality traits affect how people perceive and react to stressful events, particularly those occurring at work (Judge et al., 2002). In Hengartner et al’s. (2017) study, following an emergency evacuation that was stressful, people high in agreeableness and conscientiousness engaged in more social activities–suggesting positive appraisals of a stressful situation. They also found neuroticism was associated with fear, traumatic distress, and maladaptive coping after the stressful event. Neuroticism is also related to feelings of exhaustion at work more generally (Bakker et al., 2006). In fact, neuroticism is so intimately linked with how individuals experience discrimination that changes in neuroticism in response to perceived discrimination is thought to be one of the pathways linking discrimination to worse health and well-being later in life (Huebner et al., 2005; Sutin et al., 2016). Overall, these studies suggest that Big Five personality traits could be potential moderators of the negative influence of stressful events, such as workplace discrimination, on health and well-being. We hypothesized that neuroticism would likely increase the harmful effects of perceived discrimination on health. We also hypothesized that the remaining four traits would likely decrease the harmful effects of discrimination on health.

#### Optimism

Optimism is the general expectation that good things will happen in one’s life. Optimism is negatively associated with depressive symptoms and positively associated with life satisfaction (Chang, 1998). In a study conducted by Chang (1998), optimism was shown to enhance adjustment and reduce the association between stress and psychological well-being. Increases in optimism are also associated with higher self-rated health and fewer chronic illness over time among older adults (Chopik et al., 2015). Optimists have better physiological adjustment to stress, suggesting that optimism might enhance health through the ways individuals deal with stress (Puig-Perez et al., 2015). Across several studies, optimism has been shown to be related to well-being and a lower incidence of stress-related diseases (e.g., metabolic syndrome, cancer, and cardiovascular diseases; Friedman et al., 1992; Cohen et al., 2010; Nabi et al., 2010).

Aside from its role in influencing health, optimism also affects workplace outcomes. Optimism is a large determinant of happiness, sense of purpose, and relationships with other people at work (Malik, 2013). Optimistic people use more problem-focused strategies when dealing with stress (Norman et al., 1995). However, if problem-focused copying strategies are not effective or possible, optimistic people are more likely to choose adaptive emotion-focused copying strategies such as humor and positive reframing of the situation (Ahmed, 2015). Optimism was also found to be significantly positively correlated to job satisfaction (Ahmed, 2015). As a result, we hypothesized that optimism would buffer against the stress and negative effects of workplace discrimination.

#### Perceived Control

Perceived control is a belief in one’s capability of accomplishing or succeeding in a specific situation. Judgement of one’s efficacy determines how much effort an individual would exert when faced with challenges, and how long they persist in the face of hardships (Bandura, 1982). Perceived control in workplace is often demonstrated in individuals’ higher expectations of their job performance. People who are high in personal control are less affected by perceived weight discrimination at work, in terms of job satisfaction, attitudes, and organization commitment (Randle, 2012; Douglass et al., 2017).

People with high perceived control are not only more likely to take a proactive approach toward stressful situations at work, but they are also more likely to cope with illness and regulate health-related behaviors (O’Leary, 1992). For example, among patients with chronic illnesses, higher control and confidence in their coping ability was associated with faster recovery from the illness (O’Leary, 1992). Perceived control is associated with better overall health in older adulthood (Drewelies et al., 2017). Another study found positive relationship between increases in perceived control and immune system functioning, suggesting that it may play a protective role for physical health (Wiedenfeld et al., 1990). Indeed, among all the moderators examined here, perceived control has been most often linked to the association between perceived discrimination and health, whether it be as a moderator or a mediator of the association (Moradi and Hasan, 2004; Landry and Mercurio, 2009; Watkins et al., 2011; Douglass et al., 2017). Altogether, we hypothesized that perceived control would buffer against the negative effects of workplace discrimination on health.

#### Coworker and Supervisor Support

Coworker and supervisor support are considered forms of organizational and social support. Coworkers and supervisors provide both emotional and instrumental support. Coworkers and supervisors listen sympathetically and acknowledge other people’s feelings (i.e., emotional support). Coworkers and supervisors also provide physical aid and tangible assistance by giving advice and knowledge for solving problems (i.e., instrumental support; Fenlason and Beehr, 1994). Cohen and Wills (1985) theorized that social support directly affects stress through the self-esteem-enhancing effects of social acceptance. Supervisor’s social and emotional support helps create a positive appraisal of work environments. If employees perceive their supervisors as thoughtful and considerate, appraisals of the environment and their job satisfaction will increase (Kopelman et al., 1990).

Further, perceiving a management team as supportive can reduce employee’s role stress (Babin and Boles, 1996). Supervisor support has been shown to help mitigate work stress (Kula, 2017), decrease burnout (Brown and O’Brien, 1998) and increase job satisfaction (Eisenberger et al., 1986). In Redman and Snape (2006), work-based social support had a positive effect on job and life satisfaction, the power and prestige employees felt about their job, and affective and normative commitment. Supervisor support also helped individuals manage their appraisal of discrimination experiences at work. More broadly, such factors contributing to a positive workplace climate and perceived support have been suggested as modifying factors of the links between perceived discrimination, stress, and health (Stainback et al., 2011; Sloan, 2012; Okechukwu et al., 2014). As a result, we hypothesized that coworker and supervisor support would buffer against the negative effects of workplace discrimination on health.

## The Current Study

Perceived discrimination is associated with stress, work-related devaluation, and engaging in negative health behaviors. Moderators like Big Five personality traits, perceived control, optimism, and coworker and supervisor support influence how individuals cope with stress and are related to more positive work outcomes (e.g., job satisfaction and organizational commitment). In the current study, we examined the moderating effects of Big Five personality traits, perceived control, optimism, and coworker and supervisor support on the relationship between workplace discrimination and health over time. Specifically, we examined how discrimination affects depression, self-rated health, and the incidence of chronic illnesses in over 5,000 people followed over a six-year period from the Health and Retirement Study.

## Materials and Methods

### Participants

The study sample consisted of 5,023 working participants (*M*_*age*_ = 60.15, *SD* = 8.19; 56.1% were women) from the Health and Retirement Study (HRS). The HRS is a nationally representative and prospective panel study that has surveyed more than 22,000 Americans aged 50 + (and their partners, who may be younger) every two years (Sonnega et al., 2014). Data have been collected since 1992. The University of Michigan’s Institute for Social Research is responsible for the study and provides extensive documentation about the protocol, instrumentation, sampling strategy, and statistical weighting procedures. Regarding race/ethnicity, 72.1% identified as white, 14.7% identified as black, 9.9% identified as Hispanic/Latino, and 3.4% identified as mixed race/other. Participants averaged 13.54 (*SD* = 2.77) years of education. The average tenure at their current job was 12.67 years (*SD* = 11.60). The most common occupations were office/admin support (16.9%), sales (10.4%), and management (10.2%). The most common industries were health care and social assistance (17.4%), education (11.1%), and manufacturing (10.7%).

Data from the current study come from the 2008, 2010, 2012, and 2014 waves of data collection. In 2006, a random 50% of HRS respondents were selected and then visited for an enhanced face-to-face interview. In 2008, the remaining 50% of HRS respondents were visited for an enhanced face-to-face interview. Health and depression data were collected every two years. The psychological and work characteristics in the present study were all first assessed in 2008 (for half the sample) and 2010 (for the other half of the sample). Thus, two distinct cohorts were formed that had multiple assessments of health and depression, albeit technically at different waves [i.e., Cohort 1: Assessed in 2008 (wave 1), 2010 (wave 2), 2012 (wave 3), and 2014 (wave 4); Cohort 2: Assessed in 2010 (wave 1), 2012 (wave 2), and 2014 (wave 3)]. The cohorts were combined into one sample for the present analyses to increase statistical power and precision (Chopik et al., 2015). At the time that this manuscript is being written, the final cleaned release of the 2016 wave has not been made available to researchers.

The current study’s sample differed in many ways from the broader HRS sample. Compared to everyone who was excluded for not currently working or had missing data on health and depression at wave 1, participants in the current study were younger (*d* = 1.18), had more education (*d* = 0.41), had more perceived control (*d* = 0.27), were more extraverted (*d* = 0.16), more agreeable (*d* = 0.07), more conscientious (*d* = 0.27), less neurotic (*d* = 0.06), more open to experience (*d* = 0.26), more optimistic (*d* = 0.20), reported more coworker support (*d* = 0.51), more supervisor support (*d* = 0.29), were healthier (*d* = 0.49), had fewer chronic conditions (*d* = 0.69), and had lower depression (*d* = 0.28).

At α = 0.05, we had 99% power to detect effects larger than *f*^2^ = 0.004 for between subjects analyses and effects larger than *f*^2^ = 0.0004 for within subjects analyses.

Because we analyzed an existing data source, the Michigan State Institutional Review Board considered this research exempt from ethical oversight as it did not constitute human subjects research (IRB# STUDY00002967).

### Measures

#### Perceived Workplace Discrimination

Perceived work discrimination was assessed with six items adapted from a number of measures of perceived discrimination (e.g., McNeilly et al., 1996). Participants indicated the frequency with which each of the six occurrences happened. Sample items are, “How often are you watched more closely than others?” and “How often have you been unfairly humiliated in front of others at work?” Participants responded to each item on a scale ranging from 1(*never*) to 6(*almost every day*). Responses were averaged such that higher scores indicated more frequent workplace discrimination (*α* = 0.83).

#### Perceived Control

Perceived control was assessed using five items from Pearlin and Schooler (1978)’s Mastery Scale. The scale asks participants about their perceived ability to influence, control, and shape life circumstances. A sample item is, “I can do just about anything I really set my mind to do.”). Responses were provided on a 6-point scale (1 = *strongly disagree*; 6 = *strongly agree*) and averaged such that higher scores indicated higher perceived control (*α* = 0.89).

#### Personality

Big Five personality traits were measured using the MIDI personality scales (Lachman and Weaver, 1997), which is an adjective-based measure of personality. Participants indicated how well each adjective described them on a scale ranging from 1(*not at all*) to 4(*a lot*). Extraversion was measured with five items (α = 0.74; outgoing, friendly, lively, active, talkative); agreeableness was measured with five items (α = 0.78; helpful, warm, caring, softhearted, sympathetic); neuroticism was measured with four items (α = 0.71; moody, worrying, nervous, calm) conscientiousness was measured with five items (α = 0.67; organized, responsible, hardworking, careless, thorough); openness to experience was measured with seven items (α = 0.79; creative, imaginative, intelligent, curious, broad-minded, sophisticated, adventurous).

#### Optimism

Optimism was measured using the Life Orientation Test-Revised (LOT-R). Studies have shown that the LOT-R has good reliability and validity (Scheier et al., 1994; Tindle et al., 2009). A sample item is, “In uncertain times, I usually expect the best.” Participants are asked to rate the extent to which they agree with each item on a scale ranging from 1 (*strongly disagree*) to 6 (*strongly agree*). In total, six items were used to assess optimism (α = 0.75).

#### Coworker and Supervisor Support

Coworker support was assessed with a three-item measure with responses ranging from 1(*strongly disagree*) to 4(*strongly agree*) (Haynes et al., 1999). A sample item is, “My coworkers listen to me when I need to talk about work-related problems.” Responses were averaged to yield an overall index of coworker support (α = 0.90). Supervisor support was assess with a four-item measure with responses ranging from 1(*strongly disagree*) to 4(*strongly agree*) (Eisenberger et al., 2002). Responses were averaged to yield an overall index of coworker support (α = 0.93).

#### Overall Health

Self-rated health was assessed with the same single item at all waves: “Would you say your health is excellent, very good, good, fair, or poor?” Participants rated their health on a scale ranging from 1(*poor*) to 5(*excellent*). Self-rated health is a strong predictor of mortality (Idler and Benyamini, 1997; Schnittker and Bacak, 2014).

#### Chronic Health Conditions

An index of chronic health conditions (ranging from 0–8) was computed at each wave. Participants were asked to report if he or she was diagnosed (yes/no) by a physician with any of the following: (1) high blood pressure, (2) diabetes, (3) cancer or a malignant tumor of any kind, (4) lung disease, (5) coronary heart disease including heart attacks, angina, and congestive heart failure, (6) emotional, nervous, or psychiatric problems, (7) arthritis or rheumatism, and (8) stroke. Chronic illnesses were summed so higher values reflected more health problems.

#### Depression

Depressive symptoms were measured at all waves with a modified eight-item version of the Center for Epidemiological Studies Depression Scale (CES-D; Radloff, 1977). Participants responded to interview questions about whether or not (i.e., yes or no) they experienced each of eight symptoms in the past week (feeling depressed, felt everything they did was an effort, restless sleep, happiness, lonely, enjoyed life, sad, felt unmotivated). The number of dichotomous depressive symptoms was summed, with higher levels indicating higher levels of depression.

### Analytic Approach

To examine longitudinal changes in health, chronic illnesses, and depression, and whether these changes were moderated by workplace discrimination and personality characteristics, three multilevel models were constructed (i.e., one for each outcome). Specifically, we employed moderated growth curve models. Multilevel modeling allows for the analysis of the entire sample, whereas traditional regression approaches utilize listwise deletion of subjects who do not have complete data on all measures. Thus, if an individual had missing data on optimism, the effect is only estimated among people with data on this variable. Health, chronic illnesses, and depression at each wave were treated as within-subjects variables that varied over time. The linear effect of time was modeled on these within-subject observations. Workplace discrimination, perceived control, Big Five characteristics, optimism, coworker support, and supervisor support were entered as time-invariant predictors of health, chronic illnesses, and depression. Interactions between the individual and situational factors and workplace discrimination were also modeled (e.g., neuroticism × workplace discrimination predicting health). All of these variables were grand-mean centered before inclusion in the model. Betas from the model represent the effects of a one-unit increase in a continuous variable (or a group difference against a reference group for categorical variables) on the outcome of interest. Partial correlations are reported as a standardized effect size for each individual estimate.

Further, supplementary analyses tested interactions between workplace discrimination, coworker/supervisor support, and each of the personality traits and time to test whether the associations with health became stronger, weaker, or stayed the same over time in older adulthood. Participant gender (−1 = male, 1 = female), race, age (at Wave 1), and education (at Wave 1) were included as covariates.

## Results

### Bivariate Associations

Correlations and descriptive statistics are presented in Table 1. Replicating past research, perceived discrimination was associated with worse health, more chronic illnesses, and greater depression across all waves. Perceived control, extraversion, conscientiousness, (low) neuroticism, openness to experience, and optimism were most reliably associated with better mental and physical health at all waves. Coworker and supervisor support were also associated with better mental and physical health at all waves. All potential moderators (e.g., high perceived control, low neuroticism) were associated with perceiving less workplace discrimination. Women were more agreeable, conscientious, neurotic, and reported higher depression across waves. Older adults perceived less workplace discrimination, were more neurotic, and had worse physical health compared to younger adults. Older age was minimally associated with the potential moderators. Highly educated adults perceived less workplace discrimination and were conscientious, open to experience, optimistic, reported higher coworker and supervisor support, better mental and physical health, and lower neuroticism. Physical health, chronic illnesses, and depression were all correlated in intuitive directions (e.g., people with more illnesses reported worse health).

**TABLE 1:** Descriptive statistics and correlations among study variables.

	**M**	**SD**	**1**	**2**	**3**	**4**	**5**	**6**	**7**	**8**	**9**	**10**	**11**
1) Gender													
2) Age	60.15	8.19	−0.15**										
3) Education	13.54	2.77	–0.03	−0.04**									
4) Workplace discrimination	1.73	0.92	0.02	−0.23**	−0.10**								
5) Control	4.95	1.03	–0.01	−0.05**	0.03	−0.13**							
6) Extraversion	3.24	0.54	0.07	0.04**	0.03	−0.09**	0.25**						
7) Agreeableness	3.54	0.47	0.26**	<0.001	0.03*	−0.09**	0.17**	0.52**					
8) Conscientiousness	3.46	0.43	0.14**	−0.03*	0.14**	−0.10**	0.24**	0.30**	0.38**				
9) Neuroticism	2.01	0.61	0.09**	−0.12**	−0.09**	0.23**	−0.26**	−0.18**	−0.10**	−0.24**			
10) Openness	3.02	0.53	0.01	–0.01	0.24**	−0.05**	0.25**	0.53**	0.38**	0.36**	−0.21**		
11) Optimism	4.59	0.97	0.04**	0.03	0.20**	−0.24**	0.37**	0.30**	0.22**	0.28**	−0.40**	0.31**	
12) Coworker Support	3.20	0.62	–0.02	0.08**	0.16**	−0.36**	0.15**	0.14**	0.13**	0.13**	−0.17**	0.13**	0.23**
13) Supervisor support	3.02	0.73	0.00	0.13**	0.07**	−0.45**	0.15**	0.15**	0.15**	0.11**	−0.18**	0.10**	0.22**
14) Health W1	3.51	0.98	0.01	−0.03**	0.27**	−0.18**	0.19**	0.17**	0.10**	0.22**	−0.24**	0.21**	0.30**
15) Illness W1	1.47	1.25	0.01	0.30**	−0.08**	0.04**	−0.12**	−0.05**	0.02	−0.12**	0.14**	−0.07**	−0.15**
16) Depression W1	1.04	1.66	0.10**	−0.07**	−0.15**	0.23**	−0.17**	−0.13**	−0.04**	−0.15**	−0.37**	−0.12**	−0.32**
17) Health W2	3.49	0.98	0.02	−0.06**	0.25**	−0.17**	0.19**	0.17**	0.08**	0.22**	−0.22**	0.18**	0.30**
18) Illness W2	1.66	1.33	0.01	0.30**	−0.09**	0.06**	−0.12**	−0.04**	0.01	−0.12**	0.16**	−0.06**	−0.16**
19) Depression W2	1.05	1.71	0.09**	−0.07**	−0.16**	0.22**	−0.18**	−0.12**	–0.02	−0.17**	0.33**	−0.12**	−0.30**
20) Health W3	3.41	0.99	0.01	−0.05**	0.27**	−0.15**	0.17**	0.14**	0.07**	0.21**	−0.22**	0.16**	0.27**
21) Illness W3	1.83	1.38	<0.001	0.29**	−0.11**	0.07**	−0.12**	−0.04**	0.01	−0.12**	0.16**	−0.07**	−0.16**
22) Depression W3	1.07	1.70	0.10**	−0.05**	−0.15**	0.20**	−0.15**	−0.10**	–0.02	−0.12**	0.31**	−0.11**	−0.29**
23) Health W4	3.32	0.99	0.01	−0.07**	0.24**	−0.12**	0.15**	0.12**	0.03	0.20**	−0.23**	0.17**	0.26**
24) Illness W4	2.12	1.42	−0.06**	0.29**	−0.10**	0.05*	−0.09**	–0.02	0.02	−0.13**	0.16**	−0.05*	−0.13**
25) Depression W4	1.08	1.72	0.07**	<0.001	−0.17**	0.23**	−0.18**	−0.12**	–0.03	−0.14**	0.33**	−0.13**	−0.27**

	**12**	**13**	**14**	**15**	**16**	**17**	**18**	**19**	**20**	**21**	**22**	**23**	**24**

1) Gender													
2) Age													
3) Education													
4) Workplace discrimination													
5) Control													
6) Extraversion													
7) Agreeableness													
8) Conscientiousness													
9) Neuroticism													
10) Openness													
11) Optimism													
12) Coworker support													
13) Supervisor support	0.60**												
14) Health W1	0.19**	0.13**											
15) Illness W1	−0.06**	−0.03*	−0.41**										
16) Depression W1	−0.16**	−0.15*	−0.32**	0.16**									
17) Health W2	0.20**	0.14**	0.67**	−0.39**	−0.30**								
18) Illness W2	−0.06**	−0.03*	−0.42**	0.94**	0.18**	−0.43**							
19) Depression W2	−0.16**	−0.15**	−0.30**	0.19**	0.51**	−0.37**	0.21**						
20) Health W3	0.18**	0.12**	0.64**	−0.37**	−0.28**	0.68**	−0.39**	−0.32**					
21) Illness W3	−0.07**	−0.04**	−0.41**	0.89**	0.18**	−0.43**	0.94**	0.22**	−0.43**				
22) Depression W3	−0.14**	−0.12**	−0.29**	0.19**	0.47**	−0.32**	0.21**	0.51**	−0.38**	0.23**			
23) Health W4	0.16**	0.13**	0.58	−0.34**	−0.26**	0.65**	−0.36**	−0.33**	0.70**	−0.38**	−0.35**		
24) Illness W4	−0.05**	–0.01	−0.39**	0.83**	0.17**	−0.41**	0.89**	0.22**	−0.39**	0.95**	0.22**	−0.42**	
25) Depression W4	−0.17**	−0.15**	−0.34**	0.18**	0.45**	−0.35**	0.20**	0.51**	−0.36**	0.21**	0.57**	−0.40**	0.25**

*p < 0.05, **p < 0.01.

### Primary Analyses

The multi-level models for physical health (Table 2), chronic illness (Table 3), and depression (Table 4) are discussed below. The pseudo-*R*^2^ for the health (0.28), chronic illness (0.26), and depression models (0.33) suggested good model fit (Snijders and Bosker, 2012).

**TABLE 2:** Multi-level model predicting health.

	**b**	**SE**	**β**	**df**	**t**	**p**	**LB**	**UB**	***r***
Linear	–0.03	<0.01	–0.03	12501.00	–8.09	<0.001	–0.04	–0.02	–0.07
Age	–0.01	<0.01	–0.08	4225.24	–5.97	<0.001	–0.01	–0.02	–0.09
Gender	0.01	0.01	0.01	4204.90	1.18	0.24	–0.01	0.04	0.02
White	0.16	0.06	0.16	4241.31	2.61	0.01	0.04	0.29	0.04
Black	–0.12	0.07	–0.12	4239.44	–1.83	0.07	–0.26	0.01	–0.03
Latino	–0.07	0.07	–0.07	4237.56	–1.04	0.30	–0.22	0.07	–0.02
Education	0.06	0.00	0.16	4215.32	12.35	<0.001	0.05	0.07	0.19
Workplace Discrimination	–0.05	0.02	–0.05	4188.46	–3.18	<0.001	–0.08	–0.02	0.05
Control	0.05	0.01	0.05	4176.95	3.77	<0.001	0.02	0.07	0.06
Extraversion	0.17	0.03	0.09	4212.10	6.21	<0.001	0.12	0.22	0.10
Agreeableness	–0.15	0.03	–0.07	4201.22	–5.00	<0.001	–0.21	–0.09	–0.08
Conscientiousness	0.21	0.03	0.09	4221.33	6.82	<0.001	0.15	0.27	0.10
Neuroticism	–0.18	0.02	–0.11	4186.80	–8.24	<0.001	–0.22	–0.14	–0.13
Openness	0.02	0.03	0.01	4208.25	0.72	0.48	–0.03	0.07	0.01
Optimism	0.12	0.01	0.11	4191.17	8.30	<0.001	0.09	0.15	0.13
Coworker Support	0.10	0.02	0.06	4198.46	4.20	<0.001	0.06	0.15	0.06
Supervisor Support	–0.03	0.02	–0.02	4181.69	–1.13	0.26	–0.07	0.02	–0.02
Discrimination × Control	0.02	0.01	0.02	4165.54	1.36	0.18	–0.01	0.04	0.02
Discrimination × Extraversion	–0.07	0.03	–0.03	4170.68	–2.37	0.02	–0.13	–0.01	–0.04
Discrimination × Agreeableness	0.09	0.03	0.04	4145.28	2.99	0.003	0.03	0.14	0.05
Discrimination × Conscientiousness	–0.03	0.03	–0.01	4198.06	–0.98	0.33	–0.09	0.03	–0.02
Discrimination × Neuroticism	0.01	0.02	0.004	4146.67	0.35	0.73	–0.03	0.05	0.01
Discrimination × Openness	–0.03	0.03	–0.01	4167.96	–0.99	0.32	–0.08	0.03	–0.02
Discrimination × Optimism	0.01	0.01	0.01	4157.87	1.01	0.31	–0.01	0.04	0.02
Discrimination × Coworker Support	0.04	0.02	0.02	4108.59	1.93	0.06	<0.01	0.08	0.03
Discrimination × Supervisor Support	–0.01	0.02	–0.01	4119.82	–0.79	0.43	–0.05	0.02	–0.01

*Reference category for race is other.*

**TABLE 3:** Multi-level model predicting illnesses.

	**b**	**SE**	**β**	**df**	**t**	**p**	**LB**	**UB**	***r***
Linear	0.10	<0.01	0.10	9767.38	40.66	<0.001	0.09	0.10	0.38
Age	0.06	<0.01	0.46	3965.32	22.21	<0.001	0.05	0.06	0.33
Gender	0.03	0.02	0.03	3958.89	1.59	0.11	–0.01	0.07	0.03
White	0.14	0.10	0.14	3971.40	1.36	0.18	–0.06	0.34	0.02
Black	0.29	0.11	0.29	3970.48	2.57	0.01	0.07	0.50	0.04
Latino	–0.16	0.12	–0.16	3969.26	–1.35	0.18	–0.39	0.07	–0.02
Education	–0.03	0.01	–0.07	3961.36	–3.44	0.001	–0.04	–0.01	–0.05
Workplace Discrimination	0.09	0.03	0.08	3953.93	3.29	0.001	0.03	0.14	0.05
Control	–0.03	0.02	–0.03	3948.76	–1.52	0.13	–0.07	0.01	–0.02
Extraversion	–0.10	0.05	–0.05	3961.70	–2.18	0.03	–0.19	–0.01	–0.03
Agreeableness	0.20	0.05	0.09	3958.60	3.98	<0.001	0.10	0.30	0.06
Conscientiousness	–0.19	0.05	–0.08	3964.36	–3.83	<0.001	–0.29	–0.09	–0.06
Neuroticism	0.30	0.04	0.16	3952.38	7.50	<0.001	0.20	0.34	0.12
Openness	0.05	0.05	0.03	3959.87	1.05	0.30	–0.04	0.14	0.02
Optimism	–0.10	0.02	–0.09	3954.34	–3.94	<0.001	–0.14	–0.05	–0.06
Coworker support	–0.05	0.04	–0.03	3956.31	–1.22	0.22	–0.13	0.03	–0.02
Supervisor support	0.03	0.04	0.02	3950.70	0.68	0.50	–0.05	0.10	0.01
Discrimination × Control	–0.05	0.02	–0.04	3944.66	–2.10	0.04	–0.09	–0.00	–0.03
Discrimination × Extraversion	–0.01	0.05	–0.003	3949.00	–0.14	0.89	–0.10	0.09	–0.002
Discrimination × Agreeableness	–0.03	0.05	–0.01	3941.09	–0.70	0.48	–0.13	0.06	–0.01
Discrimination × Conscientiousness	0.07	0.05	0.03	3956.75	1.31	0.19	–0.03	0.17	0.02
Discrimination × Neuroticism	0.03	0.03	0.02	3940.26	0.83	0.41	–0.04	0.09	0.01
Discrimination × Openness	0.07	0.05	0.03	3947.49	1.45	0.15	–0.02	0.16	0.02
Discrimination × Optimism	0.02	0.02	0.01	3944.48	0.66	0.51	–0.03	0.06	0.01
Discrimination × Coworker Support	–0.01	0.03	–0.01	3928.08	–0.40	0.69	–0.08	0.05	–0.01
Discrimination × Supervisor Support	–0.01	0.03	–0.01	3931.15	–0.25	0.80	–0.07	0.05	–0.004

*Reference category for race is other.*

**TABLE 4:** Multi-level model predicting depression.

	**b**	**SE**	**β**	**df**	**t**	**p**	**LB**	**UB**	**r**
Linear	0.01	0.01	0.01	12440.94	1.00	0.32	–0.01	0.02	0.01
Age	<0.01	<0.01	0.03	4482.22	1.32	0.19	<0.01	0.01	0.02
Gender	0.11	0.02	0.11	4444.04	5.46	<0.001	0.07	0.14	0.08
White	–0.15	0.10	–0.15	4479.00	–1.54	0.13	–0.35	0.04	–0.02
Black	0.27	0.10	0.27	4479.12	2.46	0.01	0.05	0.48	0.04
Latino	0.16	0.11	0.16	4485.70	1.37	0.17	–0.07	0.38	0.02
Education	–0.05	0.00	–0.13	4468.59	–6.61	<0.001	–0.06	–0.03	–0.10
workplace discrimination	0.15	0.03	0.14	4424.11	5.90	<0.001	0.10	0.20	0.09
Control	–0.04	0.02	–0.04	4419.85	–1.86	0.06	–0.08	<0.01	–0.03
Extraversion	–0.19	0.04	–0.10	4446.62	–4.43	<0.001	–0.28	–0.10	–0.07
Agreeableness	0.22	0.05	0.10	4452.53	4.57	<0.001	0.13	0.31	0.07
Conscientiousness	–0.14	0.05	–0.06	4480.11	–2.82	0.01	–0.23	–0.04	–0.04
Neuroticism	0.63	0.03	0.38	4439.91	18.50	<0.001	0.56	0.70	0.27
Openness	0.08	0.04	0.04	4449.34	1.74	0.08	–0.01	0.16	0.03
Optimism	–0.24	0.02	–0.23	4431.85	–10.72	<0.001	–0.28	–0.20	–0.16
coworker support	–0.05	0.04	–0.03	4436.21	–1.16	0.25	–0.12	0.03	–0.02
Supervisor support	–0.01	0.04	–0.01	4418.37	–0.32	0.75	–0.08	0.06	–0.005
Discrimination × Control	0.01	0.02	0.01	4414.35	0.62	0.54	–0.03	0.05	0.01
Discrimination × Extraversion	0.07	0.05	0.03	4367.99	1.48	0.14	–0.02	0.16	0.02
Discrimination × Agreeableness	–0.05	0.05	–0.02	4375.65	–1.2	0.23	–0.14	0.03	–0.02
Discrimination × Conscientiousness	–0.00	0.05	–0.001	4454.12	–0.07	0.95	–0.10	0.09	–0.001
Discrimination × Neuroticism	0.15	0.03	0.08	4385.48	4.60	<0.001	0.08	0.21	0.07
Discrimination × Openness	–0.03	0.04	–0.01	4395.65	–0.61	0.54	–0.11	0.06	–0.01
Discrimination × Optimism	–0.05	0.02	–0.04	4379.18	–2.00	0.05	–0.09	<0.01	–0.03
Discrimination × Coworker Support	–0.03	0.03	–0.02	4317.28	–1.08	0.28	–0.10	0.03	–0.02
Discrimination × Supervisor Support	0.03	0.03	0.02	4335.10	1.19	0.23	–0.02	0.09	0.02

*Reference category for race is other.*

**FIGURE 1:**
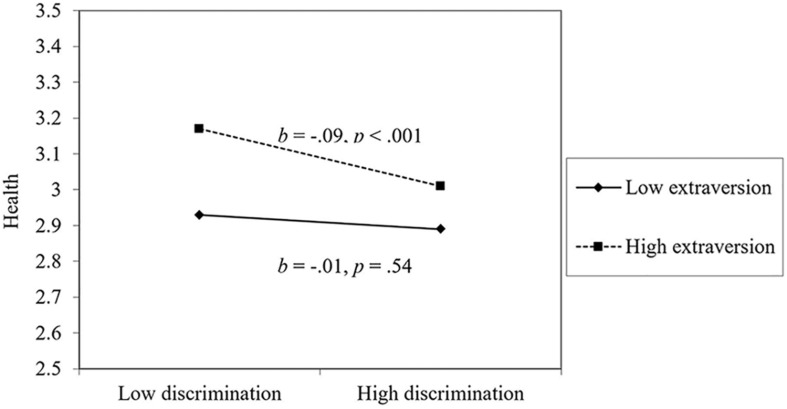
The moderating effect of extraversion on the link between discrimination and health.

**FIGURE 2:**
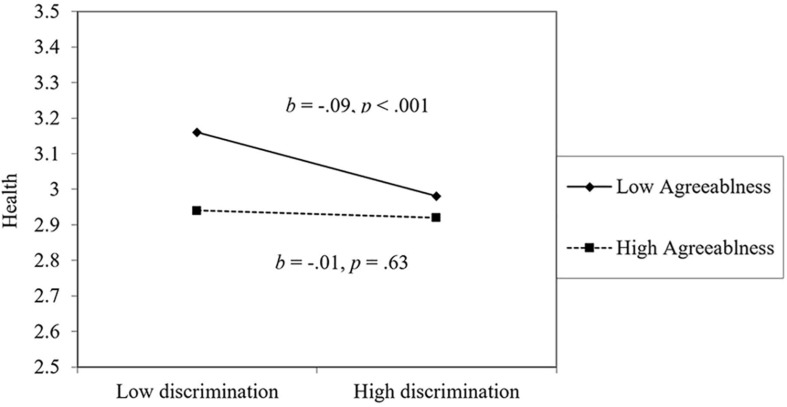
The moderating effect of agreeableness on the link between discrimination and health.

For physical health, perceiving less workplace discrimination, younger age, more education, higher perceived control, higher extraversion, lower agreeableness, higher conscientiousness, lower neuroticism, higher optimism, and more coworker support were all associated with better health. For the most part, the effect of workplace discrimination on health was not moderated by personality or workplace support, with two exceptions: extraversion and agreeableness. As seen in Figure 1, extraverts were most affected by workplace discrimination: people low in extraversion were relatively unaffected by workplace discrimination. However, worth noting, even highly extraverted people were healthier than introverts when experiencing workplace discrimination. As seen in Figure 2, people low in agreeableness were healthier, particularly when not facing workplace discrimination. This surprising finding will be examined in the Discussion. Physical health declined over time on average.

**FIGURE 3:**
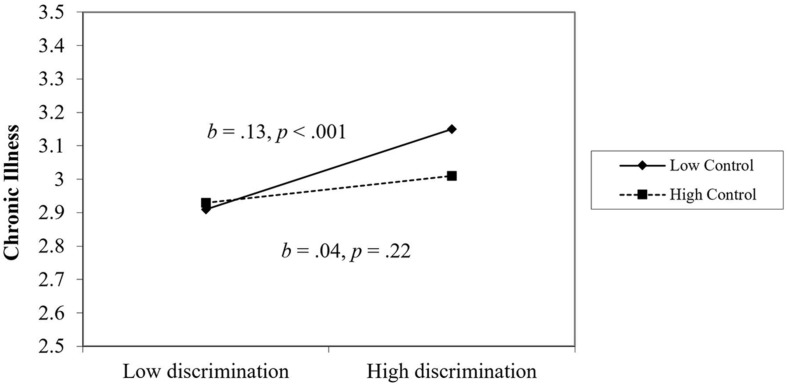
The moderating effect of perceived control on the link between discrimination and chronic illness.

For chronic illnesses, perceiving less workplace discrimination, younger age, more education, higher extraversion, lower agreeableness, higher conscientiousness, lower neuroticism, and higher optimism were associated with fewer chronic illnesses. For the most part, the effect of workplace discrimination on chronic illnesses was not moderated by personality or workplace support, with one exception: perceived control. As seen in Figure 3, workplace discrimination was particularly harmful for individuals low in perceived control. Among those high in perceived control, perceived workplace discrimination was unrelated to chronic illnesses. The number of chronic illnesses increased over time on average.

**FIGURE 4:**
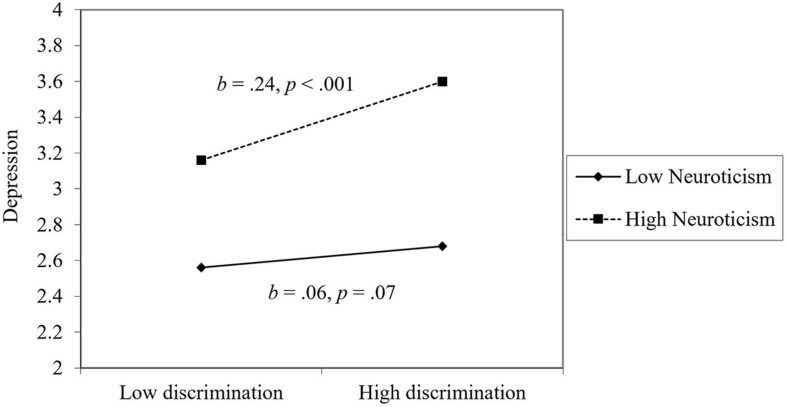
The moderating effect of neuroticism on the link between discrimination and depression.

For depression, perceiving less workplace discrimination, being male, more education, higher extraversion, lower agreeableness, higher conscientiousness, lower neuroticism, and more optimism were associated with lower depression. For the most part, the effect of workplace discrimination on depression was not moderated by personality or workplace support, with one exception: neuroticism. As seen in Figure 4, workplace discrimination was particularly harmful for individuals high in neuroticism. Among people low in neuroticism, workplace discrimination was unrelated to depression.^1^

## Discussion

The present study examined longitudinal changes in health, chronic illnesses, and depression, and whether these changes were moderated by perceived workplace discrimination and personal and situational characteristics. In this study, a number of variables (e.g., high perceived control, low neuroticism, high optimism, more coworker and supervisor support) were associated with perceiving less workplace discrimination. A number of our hypotheses were supported—extraversion, conscientiousness, optimism, perceived control (for health), and coworker support (for health) were associated with better health and well-being; perceived workplace discrimination and neuroticism were associated with worse health and well-being. Agreeableness, openness to experience, and supervisor support were either unrelated or related in the opposite way to what was hypothesized. These variables occasionally moderated associations between perceived discrimination and each outcome. This study not only confirms a number of results of previous studies but constituted an exhaustive examination of several moderating variables. It also provides insights into how workers and organizations can prevent, adjust, and react to perceived workplace discrimination.

The results of the current study align well with previous research and theory on coping in the face of discrimination and stigma (Berjot and Gillet, 2011). As Pascoe and Smart Richman (2009) state, perceived discrimination has been linked with worse health behaviors and more health problems. Our study found that workplace discrimination in particular is also associated with lower overall health, more chronic illness, and greater depression. The current study showed that highly neurotic people were more affected by workplace discrimination than those low in neuroticism—people high in neuroticism reported greater depressive symptoms in the context of perceiving workplace discrimination. Individuals high in neuroticism often experience more stressful events, respond more negatively to these stressful events, and are more likely to adopt maladaptive coping strategies in response to stressful situations (Afshar et al., 2015; Hengartner et al., 2017).

The current study also found that people with high perceived control were less negatively affected by workplace discrimination when reporting on their overall health. These findings align with previous studies showing that people high in control were more likely to take on adaptive coping strategies and take a proactive approach toward navigating difficulty situations at work (Bandura, 1982; Randle, 2012; Afshar et al., 2015). People high in perceived control are likely to be healthier because they employ these strategies when encountering stressful situations; people low in perceived control may find it difficult to be resilient in the face of workplace discrimination.

One surprising finding from the current study was that agreeableness was associated with worse health, more chronic illnesses, and greater depression. These results may have arisen from two sources. First, it could be that agreeableness is not a protective factor (and may even be a risk factor) in the context of stressful situations, like perceived discrimination. For example, agreeable individuals report more distress in response to interpersonal conflict than others (Suls et al., 1998). This might be because of their heightened sensitivity to negative interpersonal situations. This greater distress could translate to worse physical and mental health. Thus, people high in agreeableness may not be employing the most adaptive coping mechanisms at work. The same can be said for why extraverts were also negatively affected by workplace discrimination. However, there are also several studies that suggest agreeable people are healthier and utilize adaptive coping styles (e.g., Booth-Kewley and Vickers, 1994; Afshar et al., 2015; Hengartner et al., 2017). A second possibility is that these associations may have resulted from suppression effects. Supporting this interpretation are null (or very small, intuitive) bivariate associations between agreeableness and health outcomes (see Table 1). Suppression effects often do not replicate, so we encourage future researchers to examine the link between agreeableness and mental and physical health in large studies of older adults.

Based off the findings of the present study, combined with previous research, perceived workplace discrimination is directly associated with worse health and well-being. The significant moderation effects of extraversion, neuroticism, and perceived control on the link between perceived workplace discrimination and health have great significance for organizations that struggle with the effects of incivility. Maintaining a diverse and open-minded culture likely helps with lowering the prevalence of discrimination at work (Gelfand et al., 2007; Chrobot-Mason and Aramovich, 2013). Based on the results of the current study, encouraging a greater sensitivity for how workplace discrimination might be particularly harmful for some individuals (e.g., those high in neuroticism; low in perceived control) is also a worthwhile endeavor. Aside from cultivating environments that make discrimination less likely, it may be possible to cultivate skills and abilities in employees as well—so that the negative effects of perceived discrimination might sting less. Although the work on volitional personality change (and whether broader organizations can facilitate these changes) is in its infancy (Hudson et al., 2012, 2018), cultivating characteristics of perceived control and emotional stability could enhance employee well-being in the context of efforts to reduce discrimination in workplace settings.

### Limitations and Future Directions

The study had many strengths. We used a large sample of workers who completed measures of mental and physical health at multiple time points over a six-year period. Our study was also one of the most comprehensive examinations of possible moderators to date—simultaneously considering nine different buffering effects of personality and workplace environment constructs.

Nevertheless, the study had limitations that are worth noting. First, participants were older adults (*M*_*age*_ = 60.15). This limited range of age excludes younger workers and the negative effects that discrimination might have on their lives. Future research should include a wider range of participants to examine the effects of discrimination on health and whether the moderators of this link also vary by age. Further, only 27.9% of the sample was non-white. The lack of representation from people of color might have affected the results as racial discrimination may have been a major source of discrimination for these participants. Related, we hope that researchers will also employ multi-informant reports and other measures of discrimination in the future. In the current study, we focused on *perceived* discrimination from the perspective of the aggrieved party. Of course, from one perspective, it is possible that perceiving discrimination (even when no ill-intent was present) is likely more closely related to mental and physical health outcomes than discrimination measured a different way (i.e., the perception still causes stress and deleterious behaviors for an individual). However, having more nuanced and multi-faceted assessments of workplace environments (particularly with respect to discrimination) would help elucidate the effects of discrimination on health and well-being over time. Future research should try to achieve a better balance of race/ethnicity when examining questions related to discrimination.

Additionally, the current study examined a specific set of variables—Big Five personality traits, optimism, perceived control, and workplace social support. However, less is known about *why* particular factors facilitate workplace discrimination to be more toxic for health. For example, highly neurotic people report more depression in response to workplace discrimination. There could be several reasons for this. Neuroticism might be associated with greater stress reactivity or affect people’s thoughts about their workplace environment. Neuroticism might be associated with worse health behavior (e.g., substance abuse, lower physical activity) which might be affecting health in indirect ways. It is important that future research investigate the exact pathways through which individual difference characteristics put people at heightened risk (or resilience) when experiencing workplace discrimination.

## Conclusion

The current study examined the link between perceived workplace discrimination and health, along with several moderators of this link (i.e., Big Five personality, perceived control, optimism, and coworker, and supervisor support). Workplace discrimination was associated with worse health, more chronic illness, and higher depression. Extraversion, agreeableness, perceived control, and neuroticism were all implicated in either amplifying or suppressing the negative effects of discrimination on mental and physical health. The current study provides a valuable step in examining the individual and workplace characteristics that put individuals at greater risk of experiencing negative outcomes in response to workplace discrimination. Although the current study provided a descriptive account of moderators of the discrimination-health link, future applied researchers can look for actionable ways to reduce workplace discrimination and the particularly negative effects that it has on certain people.

## Data Availability Statement

Data from the Health and Retirement Study is publicly available for researchers. The study can be accessed via https://hrs.isr.umich.edu/.

## Ethics Statement

The studies involving human participants were reviewed and approved by the Michigan State Institutional Review Board. The patients/participants provided their written informed consent to participate in this study.

## Author Contributions

YX and WC conceived of this study. WC analyzed the data, created tables and figures, and provided the critical edition. YX drafted the initial manuscript.

## Conflict of Interest

The authors declare that the research was conducted in the absence of any commercial or financial relationships that could be construed as a potential conflict of interest.

## Footnotes

1 We additionally tested whether the aforementioned moderation effects increased or decreased in magnitude across the study window. To do so, we took the models found in Tables 2–4 and, for the nine two-way interactions, we computed three-way interactions with time (e.g., discrimination × perceived control × linear time). The vast majority of these effects were not moderated by time. However, three effects emerged as significant. For self-rated health, a three-way interaction between discrimination × optimism × time emerged as significant (*b* = 0.01, *t* = 2.20, *p* = 0.028). Decomposing this interaction revealed that the two-way interaction between discrimination and optimism grew over time (time 1: *b* = −0.01, *p* = 0.54; time 2: *b* = 0.02, *p* = 0.23; time 3: *b* = 0.04, *p* = 0.028; time 4: *b* = 0.02, *p* = 0.58). However, the effect was not significant at times 1, 2, and 4, so we hesitate to devote significant space to discussing this effect. For depression, three-way interactions between discrimination × extraversion × time (*b* = 0.04, *t* = 2.25, *p* = 0.025) and discrimination × openness × time (*b* = −0.04, *t* = -2.46, *p* = 0.014) emerged as significant. Decomposing the discrimination × extraversion × time interaction revealed that the two-way interaction between discrimination and extraversion grew over time (time 1: *b* = −0.0001, *p* = 0.99; time 2: *b* = −0.03, *p* = 0.62; time 3: *b* = 0.18, *p* = 0.005; time 4: *b* = 0.16, *p* = 0.13). However, the effect was not significant at times 1, 2, and 4, so we hesitate to devote significant space to discussing this effect. Decomposing the discrimination × openness × time interaction revealed that the two-way interaction between discrimination and openness became more negative over time (time 1: *b* = 0.06, *p* = 0.30; time 2: *b* = 0.03, *p* = 0.65; time 3: *b* = −0.11, *p* = 0.09; time 4: *b* = −0.26, *p* = 0.01). However, the effect was not significant at times 1, 2, and 3. At time 4, the detrimental effect of workplace discrimination on depression was stronger for those low in openness to experience and not present among those higher in openness to experience. Worth noting, the models included 47 estimates altogether (including the intercepts), so the results should be interpreted with caution given the increased likelihood for false positives. Further, many of these effects would not survive even a liberal *p*-value correction. Thus, altogether the moderating effects of personality and workplace support were largely invariant over time, suggesting that they were just as strong at time 1 as they were at time 4.

## References

[B1] Afshar H. Roohafza H. R. Keshteli A. H. Mazaheri M. Feizi A. Adibi P. (2015). The association of personality traits and coping styles according to stress level. *J. Res. Med. Sci.* 20 353–358. 26109990 PMC4468450

[B2] Ahmed M. A. (2015). The role of self-esteem and optimism in job satisfaction among teachers of private universities in Bangladesh. *Asian Bus. Rev.* 1 114–120. 10.18034/abr.v1i2.129

[B3] Babin B. J. Boles J. S. (1996). The effects of perceived co-worker involvement and supervisor support on service provider role stress, performance and job satisfaction. *J. Retail.* 72 57–75. 10.1016/s0022-4359(96)90005-6

[B4] Bakker A. B. Van der Zee K. I. Lewig K. A. Dollard M. F. (2006). The relationship between the Big Five personality factors and burnout: a study among volunteer counselors. *J. Soc. Psychol.* 146 31–50. 10.3200/socp.146.1.31-50 16480120

[B5] Bandura A. (1982). Self-efficacy mechanism in human agency. *Am. Psychol.* 37 122–147. 10.1037/0003-066x.37.2.122

[B6] Bennett G. G. Wolin K. Y. Robinson E. L. Fowler S. Edwards C. L. (2005). Perceived racial/ethnic harassment and tobacco use among African American young adults. *Am. J. Public Health* 95 238–240. 10.2105/ajph.2004.037812 15671457 PMC1449159

[B7] Berjot S. Gillet N. (2011). Stress and coping with discrimination and stigmatization. *Front. Psychol.* 2:33. 10.3389/fpsyg.2011.00033 21713247 PMC3110961

[B8] Booth-Kewley S. Vickers R. R. Jr. (1994). Associations between major domains of personality and health behavior. *J. Pers.* 62 281–298. 10.1111/j.1467-6494.1994.tb00298.x 7965560

[B9] Brondolo E. Libby D. J. Denton E.-G. Thompson S. Beatty D. L. Schwartz J. (2008). Racism and ambulatory blood pressure in a community sample. *Psychosom. Med.* 70 49–56. 10.1097/psy.0b013e31815ff3bd 18158368

[B10] Brown C. O’Brien K. M. (1998). Understanding stress and burnout in shelter workers. *Professional Psychol.* 29 383 10.1037/0735-7028.29.4.383

[B11] Carter E. R. Murphy M. C. (2015). Group-based differences in perceptions of racism: what counts, to whom, and why? *Soc. Pers. Psychol. Comp.* 9 269–280. 10.1111/spc3.12181

[B12] Chang E. C. (1998). Does dispositional optimism moderate the relation between perceived stress and psychological well-being?: a preliminary investigation. *Pers. Individ. Differ.* 25 233–240. 10.1016/s0191-8869(98)00028-2

[B13] Charles S. T. (2010). Strength and Vulnerability Integration (SAVI): a model of emotional well-being across adulthood. *Psychol. Bull.* 136 1068–1091. 10.1037/a0021232 21038939 PMC3059514

[B14] Chopik W. J. Kim E. S. Smith J. (2015). Changes in optimism are associated with changes in health among older adults. *Soc. Psychol. Pers. Sci.* 6 814–822. 10.1177/1948550615590199 27114753 PMC4841504

[B15] Chrobot-Mason D. Aramovich N. P. (2013). The psychological benefits of creating an affirming climate for workplace diversity. *Group Organ. Manag.* 38 659–689. 10.1177/1059601113509835

[B16] Cohen B. E. Panguluri P. Na B. Whooley M. A. (2010). Psychological risk factors and the metabolic syndrome in patients with coronary heart disease: findings from the Heart and Soul Study. *Psychiatry Res.* 175 133–137. 10.1016/j.psychres.2009.02.004 19969373 PMC2867840

[B17] Cohen S. Wills T. A. (1985). Stress, social support, and the buffering hypothesis. *Psychol. Bull.* 98:310 10.1037/0033-2909.98.2.310 3901065

[B18] Douglass R. P. Conlin S. E. Duffy R. D. Allan B. A. (2017). Examining moderators of discrimination and subjective well-being among LGB individuals. *J. Couns. Psychol.* 64:1. 10.1037/cou0000187 27929299

[B19] Drewelies J. Wagner J. Tesch-Römer C. Heckhausen J. Gerstorf D. (2017). Perceived control across the second half of life: the role of physical health and social integration. *Psychol. Aging* 32 76–92. 10.1037/pag0000143 28182499

[B20] Edwards J. R. Caplan R. D. Van Harrison R. (1998). Person-environment fit theory. *Theor. Organ. Stress* 28:67.

[B21] Eisenberger R. Huntington R. Hutchison S. Sowa D. (1986). Perceived organizational support. *J. Appl. Psychol.* 71:500.

[B22] Eisenberger R. Stinglhamber F. Vandenberghe C. Sucharski I. L. Rhoades L. (2002). Perceived supervisor support: contributions to perceived organizational support and employee retention. *J. Appl. Psychol.* 87:565. 10.1037/0021-9010.87.3.565 12090614

[B23] Fenlason K. J. Beehr T. A. (1994). Social support and occupational stress: effects of talking to others. *J. Organ. Behav.* 15 157–175. 10.1891/0886-6708.VV-D-17-00059 30567770 PMC6309334

[B24] Fischer A. R. Shaw C. M. (1999). African Americans’ mental health and perceptions of racist discrimination: the moderating effects of racial socialization experiences and self-esteem. *J. Couns. Psychol.* 46 395–407. 10.1037/0022-0167.46.3.395

[B25] Fleeson W. (2004). Moving personality beyond the person-situation debate:the challenge and the opportunity of within-person variability. *Curr. Direct. Psychol. Sci.* 13 83–87. 10.1111/j.0963-7214.2004.00280.x

[B26] Folkman S. Lazarus R. S. Dunkel-Schetter C. DeLongis A. Gruen R. J. (1986). Dynamics of a stressful encounter: cognitive appraisal, coping, and encounter outcomes. *J. Pers. Soc. Psychol.* 50:992. 10.1037/0022-3514.50.5.992 3712234

[B27] Friedman L. C. Nelson D. V. Baer P. E. Lane M. Smith F. E. Dworkin R. J. (1992). The relationship of dispositional optimism, daily life stress, and domestic environment to coping methods used by cancer patients. *J. Behav. Med.* 15 127–141. 10.1007/bf00848321 1583677

[B28] Gelfand M. Nishii L. Raver J. Schneider B. (2007). *Discrimination in Organizations: An Organizational-Level Systems Perspective (CAHRS Working Paper# 07-08).* Ithaca, NY: CAHRS.

[B29] Graves L. M. Powell G. (2008). *Sex and Race Discrimination in Personnel Decisions.* Oxford: Oxford Handbooks Online.

[B30] Haynes C. E. Wall T. D. Bolden R. I. Stride C. Rick J. E. (1999). Measures of perceived work characteristics for health services research: test of a measurement model and normative data. *Br. J. Health Psychol.* 4 257–275. 10.1348/135910799168614

[B31] Hengartner M. P. van der Linden D. Bohleber L. von Wyl A. (2017). Big five personality traits and the general factor of personality as moderators of stress and coping reactions following an emergency alarm on a Swiss university campus. *Stress Health* 33 35–44. 10.1002/smi.2671 26877146

[B32] Holland J. L. (1997). *Making Vocational Choices: a Theory of Vocational Personalities and Work Environments.* Odessa, FL: Psychological Assessment Resources, Inc.

[B33] Hudson N. W. Briley D. A. Chopik W. J. Derringer J. (2018). You have to follow through: attaining behavioral change goals predicts volitional personality change. *J. Pers. Soc. Psychol*. 117 839–857. 10.1037/pspp0000221 30359069

[B34] Hudson N. W. Roberts B. W. Lodi-Smith J. (2012). Personality trait development and social investment in work. *J. Res. Pers.* 46 334–344. 10.1016/j.jrp.2012.03.002 22822278 PMC3398702

[B35] Huebner D. M. Nemeroff C. J. Davis M. C. (2005). Do hostility and neuroticism confound associations between perceived discrimination and depressive symptoms? *J. Soc. Clin. Psychol.* 24 723–740. 10.1521/jscp.2005.24.5.723

[B36] Idler E. L. Benyamini Y. (1997). Self-rated health and mortality: a review of twenty-seven community studies. *J. Health Soc. Behav.* 38 21–37. 10.2307/2955359 9097506

[B37] Ingram R. E. Luxton D. D. (2005). “Vulnerability-stress models,” in *Development of Psychopathology: A Vulnerability Stress Perspective*, eds Hankin B. L. Abela J. R. Z. (Thousand Oaks, CA: Sage Publications, Inc), 32–46. 10.4135/9781452231655.n2

[B38] John O. P. Naumann L. P. Soto C. J. (2008). “Paradigm shift to the integrative Big Five trait taxonomy: history, measurement, and conceptual issues,” in *Handbook of Personality: Theory and Research*, 3rd Edn, eds John O. P. Robins R. W. Pervin L. A. (New York, NY: Guilford Press), 114–158.

[B39] Judge T. A. Erez A. Bono J. E. Thoresen C. J. (2003). The core self-evaluations scale: development of a measure. *Person. Psychol.* 56 303–331. 10.1111/j.1744-6570.2003.tb00152.x

[B40] Judge T. A. Heller D. Mount M. K. (2002). Five-factor model of personality and job satisfaction: a meta–analysis. *J. Appl. Psychol.* 87 530–541. 10.1037/0021-9010.87.3.530 12090610

[B41] Kopelman R. E. Brief A. P. Guzzo R. A. (1990). “The role of climate and culture in productivity,” in *Organizational Climate and Culture*, ed. Schneider B. (San Francisco, CA: Jossey-Bass), 282–318.

[B42] Kula S. (2017). Occupational stress, supervisor support, job satisfaction, and work-related burnout: perceptions of Turkish National Police (TNP) members. *Police Pract. Res.* 18 146–159. 10.1080/15614263.2016.1250630

[B43] Lachman M. E. Weaver S. L. (1997). *The Midlife Development Inventory (MIDI) Personality Scales: Scale Construction and Scoring (Tech. Rep. No. 1).* Waltham, MA: Brandeis University.

[B44] Landry L. J. Mercurio A. E. (2009). Discrimination and women’s mental health: the mediating role of control. *Sex Roles* 61 192–203. 10.1007/s11199-009-9624-6

[B45] Lazarus R. Folkman S. (1984). *Stress, Appraisal, and Coping.* New York, NY: Springer Publishing Company.

[B46] Malik A. (2013). Efficacy, hope, optimism and resilience at workplace–positive organizational behavior. *Int. J. Sci. Res. Publ.* 3 1–4. 10.1186/1471-2458-14-685 24997007 PMC4096423

[B47] Marchiondo L. A. Gonzales E. Williams L. J. (2017). Trajectories of perceived workplace age discrimination and long-term associations with mental, self-rated, and occupational health. *J. Gerontol. Ser. B* 74 655–663. 10.1093/geronb/gbx095 28977664 PMC6460336

[B48] Martin J. K. Tuch S. A. Roman P. M. (2003). Problem drinking patterns among African Americans: the impacts of reports of discrimination, perceptions of prejudice, and “risky” coping strategies. *J. Health Soc. Behav.* 44 408–425. 14582316

[B49] McNeilly M. D. Anderson N. B. Armstead C. A. Clark R. Corbett M. Robinson E. L. (1996). The perceived racism scale: a multidimensional assessment of the experience of white racism among African Americans. *Ethn. Dis.* 6 154–166. 8882844

[B50] Moradi B. Hasan N. T. (2004). Arab American persons’ reported experiences of discrimination and mental health: $he mediating role of personal control. *J. Couns. Psychol.* 51:418 10.1037/0022-0167.51.4.418

[B51] Nabi H. Koskenvuo M. Singh-Manoux A. Korkeila J. Suominen S. Korkeila K. (2010). Low pessimism protects against stroke: the Health and Social Support (HeSSup) prospective cohort study. *Stroke* 41 187–190. 10.1161/STROKEAHA.109.565440 19892995 PMC2884028

[B52] Norman P. Collins S. Conner M. Martin R. Rance J. (1995). Attributions, cognitions, and coping styles: Teleworkers’ reactions to work-related problems 1. *J. Appl. Soc. Psychol.* 25 117–128. 10.1111/j.1559-1816.1995.tb01587.x

[B53] Okechukwu C. A. Souza K. Davis K. D. de Castro A. B. (2014). Discrimination, harassment, abuse, and bullying in the workplace: contribution of workplace injustice to occupational health disparities. *Am. J. Indus. Med.* 57 573–586. 10.1002/ajim.22221 23813664 PMC3884002

[B54] O’Leary A. (1992). Self-efficacy and health: behavioral and stress-physiological mediation. *Cogn. Ther. Res.* 16 229–245. 10.1007/bf01173490

[B55] Pascoe E. A. Smart Richman L. (2009). Perceived discrimination and health: a meta-analytic review. *Psychol. Bull.* 135:531. 10.1037/a0016059 19586161 PMC2747726

[B56] Pearlin L. I. Schooler C. (1978). The structure of coping. *J. Health Soc. Behav.* 19 2–21. 649936

[B57] Puig-Perez S. Villada C. Pulopulos M. M. Almela M. Hidalgo V. Salvador A. (2015). Optimism and pessimism are related to different components of the stress response in healthy older people. *Int. J. Psychophysiol.* 98 213–221. 10.1016/j.ijpsycho.2015.09.002 26348260

[B58] Radloff L. S. (1977). The CES-D Scale: a self-report depression scale for research in the general population. *Appl. Psychol. Measure.* 1 385–401. 10.1177/014662167700100306 26918431

[B59] Randle N. W. (2012). Can generalized self-efficacy overcome the effects of workplace weight discrimination? *J. Appl. Soc. Psychol.* 42 751–775. 10.1111/j.1559-1816.2011.00814.x

[B60] Redman T. Snape E. (2006). The consequences of perceived age discrimination amongst older police officers: is social support a buffer? *Br. J. Manag.* 17 167–175. 10.1111/j.1467-8551.2006.00492.x

[B61] Riolli L. Savicki V. Cepani A. (2002). Resilience in the face of catastrophe: optimism, personality, and coping in the Kosovo crisis. *J. Appl. Soc. Psychol.* 32 1604–1627. 10.1111/j.1559-1816.2002.tb02765.x

[B62] Scheier M. F. Carver C. S. Bridges M. W. (1994). Distinguishing optimism from neuroticism (and trait anxiety, self-mastery, and self-esteem): a reevaluation of the Life Orientation Test. *J. Pers. Soc. Psychol.* 67 1063–1078. 10.1037/0022-3514.67.6.1063 7815302

[B63] Schnittker J. Bacak V. (2014). The increasing predictive validity of self-rated health. *PLoS One* 9:e84933. 10.1371/journal.pone.0084933 24465452 PMC3899056

[B64] Sloan M. M. (2012). Unfair treatment in the workplace and worker well-being: the role of coworker support in a service work environment. *Work Occup.* 39 3–34. 10.1177/0730888411406555

[B65] Snijders T. A. B. Bosker R. J. (2012). *Multilevel Analysis: An Introduction to Basic and Advanced Multilevel Modeling*, 2nd Edn London: Sage Publications.

[B66] Sonnega A. Faul J. D. Ofstedal M. B. Langa K. M. Phillips J. W. R. Weir D. R. (2014). Cohort profile: the Health and Retirement Study (HRS). *Int. J. Epidemiol.* 43 576–585. 10.1093/ije/dyu067 24671021 PMC3997380

[B67] Stainback K. Ratliff T. N. Roscigno V. J. (2011). The context of workplace sex discrimination: sex composition, workplace culture and relative power. *Soc. Forces* 89 1165–1188. 10.1093/sf/89.4.1165

[B68] Steffen P. R. McNeilly M. Anderson N. Sherwood A. (2003). Effects of perceived racism and anger inhibition on ambulatory blood pressure in African Americans. *Psychosom. Med.* 65 746–750. 10.1097/01.psy.0000079380.95903.78 14508015

[B69] Suls J. Martin R. David J. P. (1998). Person-environment fit and its limits: agreeableness, neuroticism, and emotional reactivity to interpersonal conflict. *Pers. Soc. Psychol. Bull.* 24 88–98. 10.1177/0146167298241007

[B70] Sutin A. R. Stephan Y. Terracciano A. (2016). Perceived discrimination and personality development in adulthood. *Dev. Psychol.* 52 155–163. 10.1037/dev0000069 26501729 PMC4941235

[B71] Tajfel H. Turner J. C. Austin W. G. Worchel S. (1979). “An integrative theory of intergroup conflict,” in *Organizational Identity: A Reader*, eds Hatch M. J. Schultz M. (New York, NY: Oxford University Press), 56–65.

[B72] Tett R. P. Burnett D. D. (2003). A personality trait-based interactionist model of job performance. *J. Appl. Psychol.* 88 500–517. 10.1037/0021-9010.88.3.500 12814298

[B73] Tett R. P. Guterman H. A. (2000). Situation trait relevance, trait expression, and cross-situational consistency: testing a principle of trait activation. *J. Res. Pers.* 34 397–423. 10.1006/jrpe.2000.2292

[B74] Tett R. P. Simonet D. V. Walser B. Brown C. (2013). “Trait activation theory: applications, developments, and implications for person-workplace fit,” in *Handbook of Personality at Work*, eds Christiansen N. D. Tett R. P. (New York, NY: Routledge), 71–100.

[B75] Tindle H. A. Chang Y. F. Kuller L. H. Manson J. E. Robinson J. G. Rosal M. C. (2009). Optimism, cynical hostility, and incident coronary heart disease and mortality in the Women’s Health Initiative. *Circulation* 120 656–662. 10.1161/circulationaha.108.827642 19667234 PMC2901870

[B76] Triana M. D. C. Jayasinghe M. Pieper J. R. (2015). Perceived workplace racial discrimination and its correlates: a meta–analysis. *J. Organ. Behav.* 36 491–513. 10.1002/job.1988

[B77] Vollrath M. (2001). Personality and stress. *Scand. J. Psychol.* 42 335–347. 11547909 10.1111/1467-9450.00245

[B78] Watkins D. C. Hudson D. L. Howard Caldwell C. Siefert K. Jackson J. S. (2011). Discrimination, mastery, and depressive symptoms among African American men. *Res. Soc. Work Pract.* 21 269–277. 10.1177/1049731510385470 24436576 PMC3891046

[B79] Wiedenfeld S. A. O’Leary A. Bandura A. Brown S. Levine S. Raska K. (1990). Impact of perceived self-efficacy in coping with stressors on components of the immune system. *J. Pers. Soc. Psychol.* 59:1082. 10.1037/0022-3514.59.5.1082 2148350

[B80] Yen I. H. Ragland D. R. Greiner B. A. Fisher J. M. (1999). Workplace discrimination and alcohol consumption: findings from the San Francisco Muni Health and Safety Study. *Ethn. Dis.* 9 70–80. 10355476

[B81] Yoshikawa H. Alan-David Wilson P. Chae D. H. Cheng J.-F. (2004). Do family and friendship networks protect against the influence of discrimination on mental health and HIV risk among Asian and Pacific Islander gay men? *AIDS Educ. Prevent.* 16 84–100. 10.1521/aeap.16.1.84.27719 15058713

